# First report on detection of *Babesia* spp. in confiscated Sunda pangolins (*Manis javanica*) in Thailand

**DOI:** 10.14202/vetworld.2021.2380-2385

**Published:** 2021-09-13

**Authors:** Rungrueang Yodsheewan, Manakorn Sukmak, Bencharong Sangkharak, Nongnid Kaolim, Raveewan Ploypan, Wallaya Phongphaew

**Affiliations:** 1 Department of Pathology, Faculty of Veterinary Medicine, Kasetsart University, Bangkhen Campus, Bangkok, Thailand; 2 Department of Farm Resources and Production Medicine, Faculty of Veterinary Medicine, Kasetsart University, Kamphaeng Saen Campus, Nakhon Pathom, Thailand; 3 Kamphangsaen Veterinary Diagnostic Center, Faculty of Veterinary Medicine, Kasetsart University, Kamphaeng Saen, Nakhon Pathom, Thailand; 4 Department of National Parks, Wildlife and Plant Conservation, Bangkok, Thailand.

**Keywords:** *Babesia* spp, *Manis javanica*, sunda pangolin, Thailand

## Abstract

**Background and Aim:**

The Sunda pangolin (*Manis javanica*) is on the International Union for Conservation of Nature Red List of Threatened Species (critically endangered) due to high levels of illegal trafficking for its products. Thailand is one of the habitats of this species, and it has become the main hub for its illegal trafficking. Rehabilitating these captive pangolins and reintroducing them back to the wild are challenging due to the limited knowledge on their diet, management, and diseases. Hemoparasites, including *Babesia* spp., can cause important protozoal infections in both domestic and wild animals, resulting in the failure of rehabilitation and conservation programs. However, *Babesia* spp. has not been reported in pangolins. The aim of the study was to determine the prevalence of *Babesia* spp. in the Sunda pangolin of Thailand.

**Materials and Methods:**

A total of 128 confiscated Sunda pangolins from across different regions in Thailand were investigated. These pangolins had been admitted to a regional Wildlife Quarantine Center for rehabilitation before release in the forest. Routine physical examinations were conducted on the animals. We collected blood samples from each pangolin for hematological analysis and to detect *Babesia* spp. using polymerase chain reaction (PCR) targeting the partial *18s rRN*A gene.

**Results:**

*Babesia*-specific PCR detected 53 animals (41.4%) that were positive for *Babesia* spp. Blood smears were obtained from the positive samples and investigated under a light microscope to observe for trophozoites of *Babesia* spp. Examination of 40 PCR-positive and -negative samples found no significant differences between the hematological parameters of *Babesia*-positive and *Babesia*-negative samples. Eight PCR-positive samples were randomly selected and their DNA was sequenced. Seven and one of sequences match uncharacterized *Babesia* spp. with 100% and 99.2% similarity, respectively. Phylogenetic analysis demonstrated that our samples form a unique monophyletic clade along with other *Babesia* spp. detected in the wild. This clade is clearly separated from other *Babesia* spp. from small carnivores, ruminants, and rats.

**Conclusion:**

Our results provide evidence of infection of Sunda pangolins in Thailand by *Babesia* spp. These pangolins originated from different regions and had not lived together before blood collection. Thus, we suggest that the uncharacterized *Babesia* spp. found in this study constitute a new group of pangolin-specific *Babesia* spp. The prevalence of the uncharacterized *Babesia* spp. was not correlated to pangolin health. Further studies are required to characterize the genomes and phenotypes, including the morphology and pathogenicity of these protozoa. Such information will be helpful for the conservation and health management of the Sunda pangolin.

## Introduction

The Sunda or Malayan pangolin (*Manis javanica*) is classified as critically endangered on the International Union for Conservation of Nature Red List of Threatened Species [1]. It is widely distributed in several countries across Southeast Asia, including Thailand [2]. However, populations of the Sunda pangolin have been gradually decreasing because of increasing threats; therefore, this species has received considerable attention regarding its conservation. Attempts to reproduce pangolins in captivity have usually been unsuccessful because of limited knowledge of their ecology and biology. Among captive pangolins, parasite infestation is one of the most common causes of morbidity and mortality [3], and among such parasites, *Babesia* spp. is known to infect humans and several kinds of animals throughout the world [4]. *Babesia* spp. belongs to the order Piroplasmida within phylum Apicomplexa, and it is closely related to *Theileria* spp. and *Cytauxzoon* spp. [5,6]. These three genera are referred to as piroplasms due to their pear-shaped (pyriform) intraerythrocytic stages. *Babesia* spp. are transmitted by hard ticks and cause morbidity and mortality in many species of mammals, both domestic and wild [4,5,7-12]. Babesiosis is a disease that can range from being asymptomatic to lethal [4,13-17].

The previous studies have reported the detection of some hemoparasites such as *Anaplasma* spp., *Rickettsia conorii*, *Ehrlichia* spp., and *Cytauxzoon* spp. in pangolins [18,19]. Although *Babesia* spp. has not been reported in pangolins, it is possible for pangolins to carry *Babesia* spp. to the detriment of their health. Moreover, studying babesiosis in pangolins may provide important information for pangolin conservation.

This study aimed to investigate the prevalence of *Babesia* spp. in Sunda pangolins and compared the blood parameters of infected and non-infected pangolins to clarify the association between babesiosis and pangolin health.

## Material and Methods

### Ethical approval

This animal use protocol has been submitted and reviewed by the Kasetsart University Institutional Animal Care and Use Committee and found to be in accordance to the guidelines of animal care and use under the Ethical Review Board of the Office of National Research Council of Thailand (NRCT) for the conduction of the scientific research. The committee approved and permitted the animal care and use to be conducted as stated in the research study in this animal use protocol (license number ACKU62-VET-053).

### Study period and location

The study was conducted from February to December 2019. The samples were collected from three provinces from central and southern Thailand, including Sukhothai, Nakorn Nayok, and Songkhla. The samples were processed at Laboratory of Department of Pathology, Faculty of Veterinary Medicine, Kamphangsaen campus.

### Sample collection

Blood samples were collected from 128 confiscated Sunda pangolins confined in three local Wildlife Quarantine Centers in Sukhothai (A; n=40), Nakorn Nayok (B; n=38), and Songkhla (C; n=50) provinces, Thailand. Whole blood samples were collected aseptically through the tail vein using a 22-gauge needle and a 5 ml syringe. Each 5 ml sample of whole blood was mixed with ethylenediaminetetraacetic acid (EDTA) in a Vacutainer blood collection tube (BD, NJ, USA). Blood samples were kept at 4°C before polymerase chain reaction (PCR) and hematological analysis at Kasetsart University, Kamphaeng Saen Campus, Nakorn Pathom, Thailand.

### Molecular analysis

DNA was extracted using a TIANamp Blood DNA kit (TIANGEN^®^, China) following the manufacturer’s protocol. PCR to detect *Babesia* spp. was performed using Phusion High-fidelity DNA Polymerase (Thermo Scientific, MA, USA) with previously reported *Babesia* spp.-specific primers targeting the *18S rRNA* gene: BAB1w (5′-GAA CCT GGT TGA TCC TGC CAG T-3′) and BAB2w (5′-GAT CCT TCT GCA GGT TCA CCT A-3′) [20]. The PCR was performed in a Peltier thermal cycler (model PTC-200; BioRad, California, USA) programmed to run at 98°C for 30 s, followed by 40 cycles of 98°C for 30 s, 58°C for 30 s, and 72°C for 90 s, ending with a final extension of 72°C for 5 min[20]. The PCR products were visualized by 1.5% agarose with iMyRun.N (Cosmo Bio, Japan) for electrophoresis using GelStar™ Nucleic Acid gel stain (Lonza, Switzerland).

### Hematological analyses

The complete blood counts of 40 representative samples (from 10 males and 10 females that tested PCR negative and positive, respectively) were measured using a Sysmex xt-2000i automated hematology analyzer (Sysmex, Japan). Serum proteins were measured using a refractometer (Brouwland, Belgium). The EDTA-treated blood from the PCR-positive samples was smeared onto glass slides, fixed in absolute methanol, and stained with modified Wright’s stain to observe for blood parasites under a light microscope.

### Sequencing and phylogenetic analyses

We randomly selected samples from eight *Babesia* spp.-positive pangolins, ensuring that each different region was represented in these samples. The purified PCR products from these samples were sequenced by the First Base Laboratory, Shah Alam, Malaysia, using the detection primer using BigDye^®^ (Thermo Fisher Scientific) Terminator v3.1 cycle sequencing kit (Applied Biosystems, CA, USA) and an ABI PRISM3130 Automated DNA Sequencer (Ibis Biosciences, CA, USA) The different *Babesia* spp. sequences were aligned using ClustalW set to default parameters using BioEdit software (Ibis Biosciences). Phylogenetic analysis was conducted using MEGA 6 software (https://www.megasoftware.net/) using a maximum-likelihood method based on the Tamura 3-parameter model with G+I (1000 replicates of bootstrap analysis were performed).

## Results

A total of 128 blood samples were obtained from Sunda pangolins that had been confiscated from illegal traffickers and housed at three regional rehabilitation and conservation centers. PCR on blood samples using *Babesia* spp.-specific *18S rRNA* gene primers indicated that 41.4% (53/128) of the samples were positive for the presence of *Babesia* spp. Pangolins from regions A, B, and C were represented in the positive samples. The highest prevalence of *Babesia* spp. was in region A, at 65% (26/40), while prevalence values in regions B and C were 21% (8/38) and 38% (19/50), respectively (Table-1). The PCR products from eight randomly selected positive samples (8/53) were sequenced and are hereafter referred to as follows: A63, A68, A70, BMDN16, BMDN37, CASM2, CASM15, and CASM21; their GenBank accession numbers, respectively, are as follows: MZ173490, MZ173491, MZ173492, MZ173493, MZ173494, MZ173495, MZ173496, and MZ173497. None of the eight sequences from the *Babesia* spp.-positive pangolins fully matched any of the sequences in GenBank. *Babesia* spp. sequences generated in this study were most similar (95.43% identity) to *Babesia* spp. that have been reported in the mane wolf (accession number: KR107880) as well as other *Babesia* spp. isolated from other wild animals. Multiple alignments of eight 782 bp long sequences demonstrate that the *Babesia* spp. detected in this study belong to two different genotypes The eight *Babesia* spp. sequences were 99.20%–100% identical. We did not detect any coinfection by these two different genotypes in this study.

**Table 1:** Number of positive samples for each sampling region

Region	Number of samples	Number of positive samples	Percentage of positive samples
A	40	26	65% (26/40)
B	38	21	55.3% (21/38)
C	50	19	38% (19/50)

**Figure-1:**
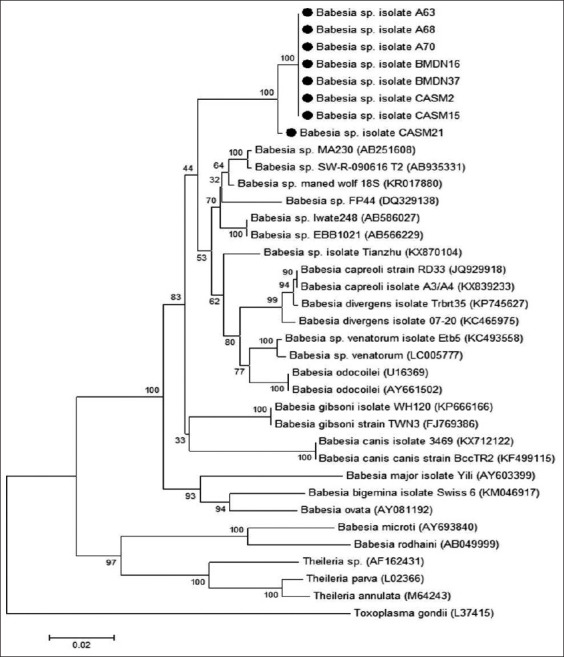
Phylogenetic analysis among *Babesia* spp. based on partial *18s rRNA* gene (782 bp). Number in brackets is GenBank accession numbers. The representative sequences of pangolin-associated *Babesia* spp. obtained in this study are marked with black circle. The phylogenetic tree was constructed using MEGA 6 [21] under a maximum-likelihood method based on Tamura 3-parameter model with G+I (1000 replicates of bootstrap analysis were performed).

A phylogenetic tree (Figure-1) was constructed based on partial *18s rRNA* sequences to verify the evolutionary relationships between pangolin-associated and other *Babesia* spp. [21]. Figure-1 shows that pangolin-associated *Babesia* spp. are segregated on a distinct branch that is monophyletically clustered with *Babesia* spp. from other wildlife (such as wolf, deer, and raccoon). This branch is clearly separated from *Babesia* spp. reported in domestic animals, small carnivores (*Babesia gibsoni* and *Brucella canis*), ruminants (*Babesia major*, *Babesia bigemina*, and *Babesia ovatus*), and rats (*Babesia microti* and *Babesia rodhaini*). The phylogenetic tree includes *Theileria* spp. sequences to provide a better understanding of the evolutionary history of this group and *Toxoplasma gondii* was used as an outgroup.

Because of the hemotropic properties of *Babesia* spp., we compared the hematological parameters of *Babesia* spp.-positive and -negative samples to investigate the association between the presence of *Babesia* spp. and the health status of Sunda pangolins. Blood samples that were positive (n=20) and negative (n=20) for *Babesia* spp. were analyzed for complete blood count and serum protein, and the results are shown in Table-2 [22]. The hematological parameters between *Babesia* spp.-positive and -negative samples did not differ significantly (p≤0.01) (Table-2). In addition, the hematological values of Babesia spp.-positive pangolins were within their normal ranges (Table-2).

**Table 2:** Comparison of hematological values between Babesia-PCR-positive and -negative Sunda pangolins, n= 20 (female = 10, male =10). No significant (p≤0.01) differences of hematological parameters were identified between PCRpositive and PCR-negative samples.

Hematological parameter	Babesia-PCR status		p-value (≤0.01)	Reference range [22]

	Babesia-positive Mean ± SD (n=20)	Babesia-negative Mean ± SD (n=20)		
RBC x 10^12^/*l*	6.31 ± 0.52	6.30 ± 0.51	0.952	1.92-9.65
HCT (%)	38.21 ± 2.85	39.33 ± 3.14	0.457	25.00-55.00
MCV (f*l*)	60.73 ± 2.05	62.57 ± 1.988	0.073	56.00-75.00
MCHC (g/*l*)	350.9 ± 7.8	350.2 ± 12.4	0.907	289.00-426.00
MCH (pg)	21.21 ± 0.82	21.89 ± 0.69	0.077	17.30-29.50
Plasma protein (g/*l*)	7.65 ± 0.71	7.86 ± 0.84	0.589	50.00-93.00
WBC Count (x 10^9^/*l*)	10.06 ± 2.18	7.49 ± 2.17	0.022	1.86-17.86
Neutrophil (x 10^9^/*l*)^a)^	6.47 ± 1.61	4.36 ± 1.78	0.018	1.29-13.96
Lymphocyte (x 10^9^/*l*)^a)^	2.37 ± 0.84	1.76 ± 0.64	0.107	0.30-3.00
Monocyte	1.17 ± 0.46	1.25 ± 0.66	0.802	0.01-2.50
Basophil	0	0	ND	0.00-0.08
Eosinophil	0.082 ± 0.07	0.12 ± 0.13	0.512	0.00-0.97
Platelet	220 ± 69.08	231.75 ± 72.49	0.755	63.00-177.00

**Figure-2:**
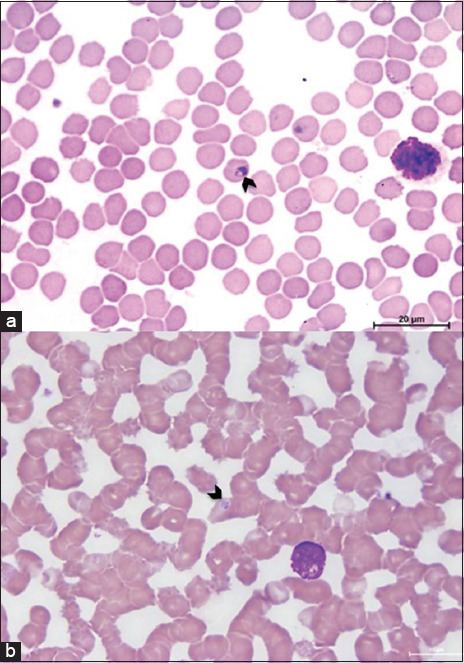
(a) Large pyriform shape intraerythrocytic trophozoite of pangolin-associated Babesia spp. (arrow head), 100×. (b) Small round shape of intraerythrocytic trophozoite of pangolin-associated Babesia spp. (arrow head), 100×.

Analysis of blood smears with modified Wright’s stain demonstrated that *Babesia* spp. are intraerythrocytic, pyriform, and round (Figure-2a and b). However, the number of *Babesia* spp. in blood smears tended to be low.

## Discussion

The results indicate that 41.4% of pangolins confiscated from illegal traffickers are infected with uncharacterized *Babesia* spp. Interestingly, these infected pangolins had the same type of *Babesia* spp., and they originated from different regions in Thailand and did not live together before blood collection. This implies that a pangolin-specific species of *Babesia* are widely distributed throughout Thailand. The results of the sequencing and phylogenetic analysis indicate that the pangolin-associated *Babesia* spp. are novel species that are genetically most similar to *Babesia* spp. reported in other wildlife. The phylogenetic tree indicates that pangolin-associated *Babesia* spp. share a common ancestor with other *Babesia* spp. detected in wildlife and are distinct from *Babesia* spp. that have been reported in domestic animals and rats. However, the pathogenic potential of the pangolin-associated *Babesia* spp. is still unknown.

The hematological analyses indicate that the hematological profiles of *Babesia* spp.-infected and non-infected pangolins are not significantly different. In addition, microscopy revealed only small numbers of *Babesia* trophozoites in blood smears. We hypothesize that *Babesia* spp. associated with the Sunda pangolin are not pathogenic to this animal, that is, the Sunda pangolin does not develop the disease caused by *Babesia* spp., but it can be infected and thus play a role as a reservoir to spread *Babesia* spp. to other wildlife. Therefore, the pangolins we observed are asymptomatic. The previous studies have observed *Amblyomma javanense*, a tick from the family Ixodidae, feeding on pangolins and other hosts such as water monitor lizards (*Varanus salvator*) and wild boar (*Sus scrofa* L.) [23-26] and thus can be a vector for circulating *Babesia* spp. in the pangolin population and other hosts. However, to date, *Babesia* spp. we described have not been reported in other hosts. Although *Babesia* spp. we described here may be potentially pathogenic to Sunda pangolins, we observed only low levels of parasitemia in infected pangolins and hematological properties did not change in response to host susceptibility factors such as age and immune status. However, all the pangolins in this study were adults, that is, no juveniles were included. The severity of illness often depends on several factors such as the specific *Babesia* species involved or the immunocompetence of the host. For example, the severity of *B. microti* infections can range from asymptomatic to fatal, and in immunocompromised persons or those that have had a splenectomy, the severity of *B. microti* infection is higher than that in healthy persons [27]. Thus, it is possible for *Babesia*-affected pangolins to develop clinical signs if their immune systems have been compromised.

Pangolin-associated *Babesia* spp. should be considered as a potential zoonotic pathogen. In general, forest encroachment, illegal trade of wildlife, and raising wild animals as pets increase the chances of humans and wildlife colocalization, thus increasing the risk of emerging zoonoses. The zoonotic potential of pangolin-associated *Babesia* spp. requires further study, particularly on the parasite’s pathogenicity and transmission cycle.

## Conclusion

We have detected a novel species of *Babesia* in Sunda pangolins. However, we did not observe any health impacts on *Babesia* spp*. -*infected pangolins, an observation that requires further study.

## Authors’ Contributions

RY and MS: Conceived the experimental design. RY and BS: Collected samples. RY, MS, BS, RP, and WP: Performed the experiment, data observation, and acquisition. RY, MS, NK, RP, and WP: Analyzed and interpreted the data. RY, MS, and WP: Wrote the manuscript. All authors reviewed the manuscript, read and approved the final manuscript.
